# The relationship of coffee consumption and CVD risk factors in elderly patients with T2DM

**DOI:** 10.1186/s12872-021-02058-7

**Published:** 2021-05-14

**Authors:** Hossein Sayed Ghavami, Mehran Khoshtinat, Sepehr Sadeghi-Farah, Arman Bayati Kalimani, Suzie Ferrie, Hossein Faraji

**Affiliations:** 1 Department of Food Science and Technology, Lahijan Branch, Islamic Azad University, Lahijan, Iran; 2 Department of Food Science and Technology, Roudehen Branch, Islamic Azad University, Roudehen, Iran; 3 Nutrition and Dietetics Department, Royal Prince Alfred Hospital, University of Sydney, Sydney, NSW Australia; 4 Department of Clinical Nutrition, School of Nutrition and Food Sciences, Isfahan University of Medical Sciences, Isfahan, Iran

**Keywords:** Coffee, Cardiovascular diseases, Diet, Elderly, Diabetes mellitus

## Abstract

**Objective:**

Clinical studies suggest increasing prevalence of cardiovascular disease (CVD) risk factors and diabetes among the elderly. Meanwhile, some food compounds, such as coffee, can also have beneficial effects on CVD risk factors. The aim of the present study was to examine the relationship between coffee consumption and CVD risk factors in the elderly with type 2 diabetes mellitus (T2DM).

**Methods:**

This cross-sectional study was performed during 2017 on 300 elderly people above 60 years of age with T2DM in Isfahan, Iran. Dietary assessment was performed using a food frequency questionnaire. Coffee consumption was classified into three groups including < 1, 1–3, and > 3 cups/day. Partial correlation test was used to investigate the relationship between CVD risk factors and usual coffee consumption.

**Results:**

The mean age and body mass index of participants were 70.04 ± 4.87 years and 24.74 ± 3.34 kg/m^2^ respectively. Coffee consumption had a significant inverse relationship with fasting plasma glucose (FPG) and diastolic blood pressure (DBP) in the elderly with T2DM (r: − 0.117, 0.134; p: 0.046, 0.022). Triglyceride (TG) and high-density lipoprotein cholesterol (HDL-C) had a significant positive relationship with coffee consumption levels (r: 0.636, 0.128; p: 0.028, 0.029). These results were obtained after controlling for potential confounders.

**Conclusion:**

Increasing coffee consumption was linked to improved status of some CVD risk factors including FPG, HDL-C, and DBP in the elderly with T2DM. Nevertheless, increasing coffee consumption was also associated with higher TG level and had no significant effect on other risk factors. Further studies are required to confirm these results.

## Introduction

Evidence suggests that there is an increase in the prevalence of diseases such as diabetes, metabolic syndrome, and cardiovascular disease (CVD) due to increased inflammation following ageing [1]. CVD has become the most important cause of mortality worldwide, with perhaps 33.7% of deaths now associated with CVD. It is estimated that by 2030, around 23.6 million deaths will occur due to CVD every year [1, 2]. CVD is known as one of the major causes of mortality in Iran [3]. One of the most important modifiable risk factors for CVD is lifestyle. Increasing physical activity, consuming a healthy diet (reducing saturated fatty acids, salt, trans fatty acids, and calorie consumption) and not smoking tobacco can lower the risk of developing CVD [4, 5]. Nevertheless, there are some specific food components such as coffee in the food basket which can also modify this equation (6, 7).

The concept of coffee as a health-related beverage has become an interesting subject of research worldwide in recent decades [8]. New evidence indicates coffee could be associated with a reduction in CVD-related mortality [9]. On the other hand, the caffeine in coffee can function as a physiological stimulus and thus result in side effects such as insulin resistance and hypertension [10]. Previous studies found that moderate coffee consumption is inversely related to CVD risk factors [11–14] but the mechanism of effect of coffee on chronic diseases and their common risk factors is not clearly known. Nevertheless, this drink has well-known antioxidant and anti-inflammatory properties which could potentially be playing a significant role in preventing CVD, cancers, neurological and psychological disorders, as well as diabetes [7, 10]. The minerals and polyphenols in coffee such as chlorogenic acid can also affect blood glucose and lipid metabolism [15]. Epidemiological studies suggest that drinking coffee can reduce some risk factors leading to death in type 2 diabetes mellitus (T2DM) as well as CVD patients [16, 17]. On the other hand, there are lifestyle factors that could possibly confound this relationship. Smoking tobacco can also result in diminished positive effects of coffee [18].

Coffee consumption varies in different populations. Population studies suggest that a high level of coffee consumption is common among elderly men and smokers [10, 19], but many of the previous studies examining the relationship between coffee consumption and CVD risk factors were conducted in industrialized countries, in populations with a wide age range [6, 7, 20]. The trend of increasing average age of Iran’s population over the recent decades, and the increasing rate of mortality due to CVD, means that older populations are of interest [21], but the number of studies in elderly populations with T2DM is limited in developing countries such as Iran. Accordingly, the aim of the present study was to explore the relationship between coffee consumption and CVD risk factors among the elderly with T2DM in Iran.

## Materials and methods

### Study population

The present study was conducted in 2017 with a cross-sectional design, targeting elderly patients under care in geriatric healthcare centers in Isfahan city, Iran. The sample selection was performed as cluster sampling; based on a sample size calculation formula (prevalence: 0.5, standard error: 0.07, design effect: 1.5, significance level: 0.05), where the target number of participants in this study was set at 300. The inclusion criteria were as follows: ability to perform daily activities, living in geriatric healthcare centers, having T2DM, and aged above 60 years. Note that T2DM diagnosis was made by a skilled medical team in reviewing patients’ files, based on the medical history, drugs taken, and laboratory data records. Next, the participants responded to the questions related to anthropometric information, diet, lifestyle, plus sociodemographic information. Blood samples were taken by a skilled physician from each subject to perform biochemical analyses.

### Dietary assessment

The typical dietary intake of each subject was assessed by an experienced dietitian using a 168-item food frequency questionnaire (FFQ). This questionnaire includes a list of typical food items consumed by Iranians. Based on the data obtained, the frequency of food consumption over the last year was also determined on a daily, weekly, and monthly basis for each subject, with the daily consumption of each food item in the last year calculated for every senior participant. Additionally, the level of coffee consumption was quantified for each subject in the last year (cup per day/week/month). Portion sizes of the food items consumed were converted to gram/day through household measurements. To calculate intake data for total calorie, macronutrients and micronutrients, nutrition IV software (Tinuviel Software, Warrington, UK) was employed.

Note that in this study, no question was asked about method of coffee preparation (espresso, filtered, instant, etc.) from the subjects. However, they responded to questions about the method of coffee consumption such as the addition of milk or sweetener. The daily coffee consumption was categorized into three groups based on standard cup size (50 ml): less than 1 cup per day (low), 1–3 cups per day (moderate), and more than 3 cups per day (high). The reliability and validity of FFQ have been demonstrated in previous studies [22]. The validation of this questionnaire was also measured following comparison of dietary intake obtained from 24-h dietary recall in 12 consecutive months (one year) with the dietary intake obtained from FFQ. The reliability of the questionnaire was also examined by comparing the food groups obtained from two separate FFQs which had been completed in one year. The results obtained from Pearson correlation statistics revealed acceptable agreement between 24-h recall in 12 consecutive months (one year) and FFQ, as well as between two FFQs (completed in one year), respectively. This questionnaire also showed notable reliability with regards to coffee consumption during a one-year period [23, 24]. Generally, the above points suggest that FFQ can also present valid assessment for coffee consumption in the long term.

### Basic characteristics

Sociodemographic and lifestyle information was obtained from patients including age, gender (male/female), smoking status (nonsmoker/current smoker), level of education (< primary school; ≥ primary school), alcohol consumption (yes/no), use of drugs (yes/no), patient-reported physical activity (low, moderate, high), and history of underlying disease (yes/no; except for T2DM). Anthropometric measurements captured weight, height, waist circumference. The weight of each patient was measured by a standard digital balance (accuracy: 100 g) with minimal clothing and barefoot). The patients’ height was measured by a measuring tape (barefoot) with accuracy of 0.5 cm, after which body mass index (BMI) was determined via wt/ht^2^ formula. Finally, waist circumference was measured using an inelastic measuring tape at the narrowest part of the torso below the ribs.

### Biochemical analyses

The blood samples of each participant were collected after 12 h of fasting. This was performed based on standard protocols and by an experienced medical team. Using a proper kit and via enzymatic-colorimetric glucose oxidase method, the fasting plasma glucose (FPG) was measured. The enzymatic-colorimetric method was utilized to measure the lipid profile including total cholesterol (TC), low-density lipoprotein (LDL-C), high-density lipoprotein (HDL-C), and triglyceride (TG). Plasma homocysteine was also measured by ELISA method and via Axis/homocysteine EIA kit (IBL Co., Germany). Finally, systolic blood pressure (SBP) and diastolic blood pressure (DBP) of patients was measured by a proper sphygmomanometer (Microlife BP AG1-20, Espenstrasse 139/ 9443/widnau/Switzerland) by a trained nursing technician.

### Statistical analysis

The variables examined in this study were described according to coffee consumption classification, and reported as number, percentage (%), and mean ± SD in relevant tables. Chi Square test was used to compare categorical variables among coffee consumption subgroups. This comparison for continuous variables was performed using one-way ANOVA and Kruskal–Wallis test. Assumption of normality was tested using the Kolmogorov–Smirnov test and homogeneity of variances was assessed by Levene’s statistic. Partial correlation was used to assess the relationship between each coffee consumption level (cups/day) and factors including energy intake, caffeine consumption, cigarette smoking, physical activity, BMI, gender, use of added sugar, history of underlying disease (except for T2DM), history of taking drugs, and level of education. All of the analyses performed in this study were done by SPSS software (version 22; Chicago, IL, USA), with the significance level considered *P* value < 0.05.

## Results

The patients participating in this study had a mean age of 70.04 ± 4.87 years, and BMI of 24.74 ± 3.34 kg/m^2^. Of these, 52.3% had BMI above 25 kg/m^2^, while 47.7% had BMI below 25 kg/m^2^. From among all participants, 55% were female and 45% were male. In addition, 93.3% of participants did not smoke, and 71.3% reported low physical activity. The mean coffee consumption among the participants was 2.43 ± 1.41 cups/day, equivalent to 121.5 mL coffee drink (1 cup: 50 mL). Note that none of the participants reported alcohol consumption. Also, all participants reported consumption of caffeinated coffee plus added white sugar. In other words, none of them reputed consumption of decaffeinated coffee. It is noteworthy that none of the subjects were receiving cholesterol-lowering therapy.

The information related to lifestyle factors, caffeine intake, measurements related to each CVD risk factor, as well as the demographic data based on daily coffee consumption are shown in Table 1. Moderate coffee consumption (1–3 cups/day) was higher among those with a high level of education (≥ primary school) compared to less-educated patients (< primary school) (*P* = 0.030). FPG and DBP were significantly lower among those consuming coffee (*P* = 0.015, 14). On the other hand, with increase in coffee consumption, HDL-C increased significantly (*P* = 0.034). Further, the blood TG was significantly lower in those reporting moderate coffee consumption (1–3 cups/day), as compared to the two other groups (*P* = 0.036). (Table 1).

**Table 1:** characteristics of elderly subjects with T2DM according to coffee consumption subgroups, Isfahan, Iran, 2017

Characteristics ^†^		Coffee intake (cups/day)				*P* value ^*^
		< 1	1–3	> 3	Total	
No. of patients, n (%)		83 (27.7)	141 (47)	76 (25.3)	300 (100)	
Age (year)		69.67 ± 4.87	70.02 ± 5.12	70.48 ± 4.37	70.04 ± 4.87	0.576^α^
Body mass index (kg/m^2^)		24.78 ± 3.23	24.93 ± 3.42	24.35 ± 3.33	24.74 ± 3.34	0.471^α^
Waist circumference (cm)		96.65 ± 4.91	96.30 ± 5.17	97.14 ± 6.24	96.61 ± 5.39	0.425^c^
Sex, n (%)	Men	36 (43.4)	68 (48.2)	31 (40.8)	135 (45)	0.541^b^
	Women	47 (56.6)	73 (51.8)	45 (59.2)	165 (55)	
Physical activity level, n (%)	Low	60 (72.3)	103 (73)	51 (67.1)	214 (71.3)	0.641^b^
	Moderate/High	23 (27.7)	38 (27)	25 (32.9)	86 (28.7)	
Smoking status, n (%)	Non smoker	76 (96.1)	131 (92.9)	73 (96.1)	280 (93.3)	0.478^b^
	Current smoker	7 (8.4)	10 (7.1)	3 (3.9)	20 (6.7)	
Educational level, n (%)	≥ Primary school	34 (41)	73 (51.8)	47 (61.8)	154 (51.3)	0.030^b^
	< Primary school	49 n	68 (48.2)	29 (38.2)	146 (48.7)	
Drug uses, n (%)	No	62 (74.7)	102 (72.3)	53 (69.7)	217 (72.3)	0.784^b^
	Yes	21 (25.3)	39 (27.7)	23 (30.3)	83 (27.7)	
Diseases history ^€^, n (%)	No	48 (57.8)	87 (61.7)	47 (61.8)	182 (60.7)	0.825^b^
	Yes	35 (42.2)	54 (38.3)	29 (38.2)	118 (39.3)	
Total energy intake (kcal/day) ^©^		2100.28 (804.19)	2130.0 (734.08)	2009.03 (884.9)	2153.33 (728.02)	0.280^α^
FPG (mg/dL)		157 ± 12.98	151.52 ± 12.18	151.09 ± 13.70	152.93 ± 13.01	0.015^c^
TG (mg/dL)		147.07 ± 22.67	140.42 ± 22.94	147.57 ± 22.98	144.07 ± 23.06	0.036^α^
TC (mg/dL)		180.51 ± 15.70	181.03 ± 15.83	180.87 ± 15.73	180.85 ± 5.72	0.972^α^
LDL-c (mg/dL)		96.36 ± 15.03	94.55 ± 15.06	96.11 ± 15	95.44 ± 15.01	0.620^α^
HDL-c (mg/dL)		47.73 ± 7.30	47.46 ± 6.93	49.92 ± 6.10	48.16 ± 6.89	0.034^α^
Homocysteine (µmol/L)		12.46 ± 2.89	12.42 ± 2.95	12.57 ± 3.52	12.47 ± 3.08	0.944^α^
SBP (mm Hg)		122.28 ± 9.88	125.05 ± 9.85	122.42 ± 10.32	123.62 ± 10.03	0.068^α^
DBP (mm Hg)		78.28 ± 10.16	77.11 ± 9.99	73.78 ± 10.02	76.59 ± 10.15	0.014^α^

T2DM: Type 2 diabetes mellitus; FPG: Fasting plasma glucose; TG: Triglyceride; TC: Total cholesterol; LDL-c: Low-density lipoprotein cholesterol; HDL-c: High-density lipoprotein cholesterol; SBP: Systolic blood pressure; DBP: Diastolic blood pressure. ^a^ One-way ANOVA test; ^b^ Chi-square test; ^c^ Kruskall-Wallis test

^€^ Except for type 2 diabetes mellitus; ^**†**^All values are expressed as mean ± standard deviation (SD) and number (percent) (except for energy intake). ^©^ expressed as median (IQR). ^*^P value < 0.05 considered as significant level

Table 2 reports the relationship between CVD risk factors and coffee consumption among the elderly with T2DM using partial correlation statistics. Results showed that with increase in coffee consumption, FPG and DBP were significantly decreased (r: −0.117; −0.134, *P* = 0.046; 0.022). On the other hand, blood TG increased significantly with increase in coffee consumption (r: 0.636; *P* = 0.028). The results obtained after adjusting for the variables of weight, BMI, gender, energy intake, caffeine consumption, history of underlying disease (other than T2DM), cigarette smoking, physical activity, added sugar, history of taking drugs, and level of education were also reported. (Table 2).

**Table 2:** Partial correlation of CVD risk factors with coffee consumption among elderly patients

Variables ®	coffee consumption (cups/day)		
	Mean ± SD	r	P^*^
FPG (mg/dL)	152.93 ± 13.01	− 0.117	0.046
TG (mg/dL)	144.07 ± 23.06	0.636	0.028
TC (mg/dL)	180.85 ± 5.72	− 0.009	0.881
LDL-c (mg/dL)	95.44 ± 15.01	− 0.006	0.916
HDL-c (mg/dL)	48.16 ± 6.89	0.128	0.029
Homocysteine (µmol/L)	12.47 ± 3.08	0.020	0.739
SBP (mm Hg)	123.62 ± 10.03	− 0.014	0.809
DBP (mm Hg)	76.59 ± 10.15	− 0.134	0.022

CVD: Cardiovascular diseases; FPG: Fasting plasma glucose; TG: Triglyceride; TC: Total cholesterol; LDL-c: Low-density lipoprotein cholesterol; HDL-c: High-density lipoprotein cholesterol; SBP: Systolic blood pressure; DBP: Diastolic blood pressure. ® Adjusted for BMI, sex, age, physical activity, added sugars, total energy intake, smoking, education level, history of diseases (except for type 2 diabetes mellitus), drug uses and intake of caffeine. * *P* value < 0.05 considered as significant level

## Discussion

The current cross-sectional study investigated the relationship between coffee consumption and CVD risk factors amongst elderly patients with T2DM. The results showed that there was an inverse relationship between coffee consumption and FPG as well as DBP levels among the elderly with T2DM. On the other hand, coffee consumption was associated with increased TG and HDL-C. These results were obtained after adjusting for potential confounders. In other words, the patients who consumed more than 3 cups of coffee per day had lower FPG and DBP, while higher HDL-C and TG.

Research examining the relationship between coffee consumption and CVD risk factors has mostly been performed among healthy individuals and with different age ranges across various countries. There are few studies assessing the relationship between coffee consumption and CVD risk factors among the elderly, who are considered as an age group with high coffee consumption [7]. The research results suggest that the effects of coffee on individuals may vary. Some of these studies suggest an inverse relationship between coffee consumption and CVD risk factors, while others have shown a direct relationship between coffee consumption and CVD risk factors [6, 25, 26]. The important point is that some clinical studies have suggested that there is an inverse relationship between coffee consumption and CVD risk factors in T2DM patients in particular [27, 28]. The results of the Nurses’ Health Study indicated that coffee consumption leads to diminished risk of developing diabetes [29]. Also, the study by Panagiotakos et al. indicated that moderate coffee consumption would reduce the risk of developing T2DM among the elderly [30]. In order to justify these discrepancies, the following points can be mentioned: type of study design, participants (healthy, patient, teenager, young, or old) as well as various dietary factors such as daily coffee consumption and different forms of coffee consumption [6].

Various risk factors have been identified for CVD, with elevated blood sugar being one of the notable factors in this regard. Studies suggest that daily coffee consumption has antidiabetic effects [31]. Coffee consumption can also affect blood glucose levels in different ways including blocking alpha- glucosidase 1 and glucose-6-phosphate translocase 1, elevating glucose-like peptide 1 (GLP-1) level and reducing the amount of glucose released from the liver [32]. Nevertheless, the effect of coffee and caffeine consumption on glucose homeostasis in the long run has remained understudied. The results of the present research indicated an inverse relationship between coffee consumption and FPG among the elderly with T2DM. In line with our results, the study by Mirmiran et al. showed that coffee consumption might significantly reduce the risk of developing T2DM [33]. On the other hand, the results by Yarmolinsky et al. suggest that coffee consumption had no significant relationship with FPG and HbA1c, but that coffee consumption can significantly lower the 2-h postload glucose level [34]. Also, Keirns et al. found that coffee consumption does not have a significant effect on FPG and glycemic response [35]. In order to justify the discrepancies across these results, the following hypotheses can be mentioned. Considering the changes in glucose homeostasis in T2DM patients as well as the altered absorption capacity for food among the elderly, the influence of coffee on blood glucose level may also be dependent on food intake and postprandial modifications [34, 36]. Further, the role of genetics in investigating the relationship between coffee and blood glucose level is also notable. Genetic diversity in the cytochrome p-450 hepatic enzyme (P450 1A1 (CYP1A2)) can also affect the relationship between coffee and FPG [37]. This enzyme has also different alleles as the main factor of coffee metabolism in the liver. Studies show that individuals with a slow allele of CYP1A2 * 1F have impairments in their FPG [38, 39].

The mechanism of effect of coffee on the lipid profile is still not fully understood. Some studies suggest that the antioxidants in coffee (e.g. chlorogenic acid, caffeic acid, trigonelline) may decrease lipid oxidation and then cause elevated TC as well as LDL-C [6, 40, 41]. On the other hand, in our study a higher coffee consumption resulted in higher TG and HDL-C but had no significant effect on TC and LDL-C. Confirming our results, the study by Alhamhany et al. showed that consumption of green coffee led to elevated HDL-C and VLDL in 35 obese individuals aged 20–55 years [42]. Similarly, in a study by Ihim et al., coffee consumption resulted in elevated HDL-C among 30 men 18–30 years of age, but had no significant effect on TG, LDL-C, and TC [43]. On the other hand, a study by Panagiotakos et al. with the aim of examining the relationship between coffee consumption and prevalence of T2DM showed that drinking coffee had no significant effect on TG, LDL-C, TC, and HDL-C. The participants in this study included 937 elderly with the age range of 65–100 years [30]. The results of Miranda et al. also suggested that coffee consumption among 557 healthy participants in São Paulo, Brazil had no significant effect on any lipid factors (TC, TG, HDL-C, and LDL-C) [6]. Method of coffee preparation might be one factor that explains the variation in results. Unfiltered coffee is packed with oily granules (diterpenes: cafestol and kahweol), which may lead to elevated cholesterol and thus higher LDL, TC, and TG [40, 44], while coffee filtration results in removal of the oil granules mentioned above, and may lead to an improved lipid profile [6, 45]. Since the participants in the present study may have used various methods of coffee preparation, the above hypothesis could be considered as a possible explanation for the mixed results.

Hypertension is known as one of the significant risk factors of CVD as well as diabetes [46]. So far, numerous studies have been conducted examining the effect of coffee consumption on blood pressure; however, their results are contradictory. The present study showed that coffee consumption had an inverse relationship with DBP but had no significant relationship with SBP among the elderly with T2DM. In this regard, the results of the study by Miranda et al. indicated that coffee consumption among 557 healthy individuals with the mean age of 45 years can significantly reduce the chance of developing high systolic or diastolic blood pressure [6]. Meanwhile, one of the clinical studies conducted on the elderly to examine the relationship between coffee and prevalence of T2DM also reported that increased coffee consumption may lead to higher SBP, but it had no effect on DBP [30], while a study by Noordzij et al.found a significant increase in both SBP and DBP with regular coffee consumption [47]. Caffeine could be a possible factor explaining the discrepant results discussed above. Evidence suggests that caffeine consumption causes significant elevation of blood pressure, but coffee consumption does not consistently have such an effect [48]. It has been suggested that this could be due to other chemical compounds in coffee, which may neutralize the effect of caffeine on elevating blood pressure. This blood pressure lowering effect may occur through vasodilation by elevating nitric oxide (NO) level and improving vascular endothelial function [49, 50]. These chemical compounds include potassium, magnesium, caffeic acid, p-coumaric acids, ferulic acid, and chlorogenic acid [51]. Since coffee consumption among the elderly population is relatively high [7], it can be concluded that coffee consumption in this group of people can also reduce blood pressure.

The present study had the following limitations: (1) previous studies evaluated both caffeinated and non-caffeinated coffee; however, all participants in our study reported only caffeinated coffee consumption, which reduces the applicability of the results. (2) the design of the present study was cross-sectional, which can increase recall biases especially in assessing dietary intake and dietary habits in the elderly. (3) alcohol intake, as a potential confounder of the results, was not reported by any participant. The possiblity that some participants did consume alcohol but did not report it due to the social pressure of the customs in Iran, mean that alcohol consumption may potentially have affected each of the outcomes examined in our study and subjected the final conclusion to bias. The strengths of this study are as follows: (1) this study has been one of the few studies based on the elderly population in Iran which examined the relationship between an important range of CVD risk factors and coffee consumption simultaneously. (2) the results of the present study have been obtained by controlling for the most important potential confounders, increasing the validity of this study.

## Conclusion

The current study indicated that increasing coffee consumption among the elderly with T2DM had a positive relationship with changes in HDL and TG, and a negative relationship with FPG and DBP, but had no effect on other CVD risk factors in these individuals. Generally, this study showed that increasing coffee consumption may reduce the major and important CVD risk factors among the elderly with T2DM. Nevertheless, further studies are required to confirm the results of the present study.

## Declarations

### Availability of data and materials

All research methods were carried out in accordance with relevant guidelines and regulations and the datasets used for this study are available from the corresponding author.
